# A Comparative Study of Abnormal Heating Composite Insulators

**DOI:** 10.3390/polym15132772

**Published:** 2023-06-21

**Authors:** Song Gao, Yunpeng Liu, Le Li

**Affiliations:** 1 Hebei Key Laboratory of Distributed Energy Storage and Micro-Grid, North China Electric Power University, Baoding 071000, China; gaosonghv23@163.com (S.G.); lile@ncepu.edu.cn (L.L.); 2 State Grid Jiangsu Electric Power Co., Ltd., Research Institute, Nanjing 211103, China

**Keywords:** abnormal heating, composite insulator, temperature characteristic, infrared thermal image

## Abstract

The abnormal heating of composite insulators on transmission lines frequently occurs, seriously threatening the power grid’s safe and stable operation. For different types of abnormal heating composite insulators, undifferentiated replacement wastes a lot of labor and material resources. This study explores the abnormal heating composite insulators under different environmental humidity and wind speed conditions. The heating and discharge of composite insulators are observed, and the heating range, heating shape, and temperature rise (Δ*T*) are analyzed. The abnormal heating of the sheath-aging composite insulator is related to the aging of the silicone rubber and the environmental humidity. The partial discharge caused by the core rod’s defect is the primary source of the temperature rise in the decay-like insulator. The heating range of the contaminated insulator is connected to the environmental humidity, and the Δ*T* increases with the salt density. The Δ*T* and heating range of the three types of abnormal heating increase with the ambient humidity. The heating phenomenon under low humidity is an important characteristic that distinguishes decay-like and contaminated insulators from sheath-aging insulators. Therefore, the on-site infrared inspection should be carried out in sunny and windless weather to prevent the impact of high humidity and wind speed on infrared temperature measurement.

## 1. Introduction

Composite insulators are increasingly used in the power grid and have become critical equipment in the transmission lines [1,2]. The reliability of the power grid is directly correlated with its performance. Therefore, the power grid operation and maintenance department pays close attention to the composite insulators. Complicated environmental, electrical, and mechanical loads—such as acid rain, pollution, and other harsh environments—affect composite insulators during operation. Furthermore, the temperature rise caused by partial discharge will further accelerate the aging of materials, seriously affecting the safe and stable operation of power equipment [3,4]. Under the combined effect of various factors, the housings of the composite insulators are gradually aging, and accidents such as pollution flashovers, abnormal heating, internal breakdown, and fracture occur [5,6,7]. Abnormal heating has been the most frequent fault in the power grid in recent years. Abnormal heating will decrease the insulation performance of composite insulators and even lead to fracture, which seriously disturbs the stability of the power system [3,8].

At present, there are many hypotheses about the mechanism of the composite insulators’ abnormal heating [9,10,11]: (1) The heating caused by partial discharge (PD) in the core rod–sheath interface; (2) Dielectric loss heating of water or moisture penetrating the sheath or interface; and (3) Resistance loss heating caused by the decrease in insulation resistance after sheath aging.

The abnormal heating of sheath-aging insulators under various humidity levels was studied by Tu et al. [12,13]. The dielectric constant and dielectric loss factor of silicone rubber (SIR) in high voltage (HV) end was also measured. These findings indicated that the aging of silicone rubber was the internal cause of abnormal heating, and the high humidity was the external cause. The sheath-aging composite insulator heated only at the HV end, and its heating was correlated with the aging sheath, the ambient humidity, and the electric field strength at the HV end. Zhang et al. [14] observed the microstructure of the end-heating composite insulator and found that the micropores on the sheath enhanced the moisture absorption and the electric field at the HV end, which increased the dielectric loss of the sheath after moisture absorption. Due to the electric field concentration at the HV end, the effect of porosity on temperature rise (Δ*T*) was more significant. Yuan et al. [15] discovered that the decay-like insulator generated heat at high (72% ± 7% RH) and low humidity (35% ± 7% RH), where RH denotes relative humidity. The Δ*T* was significantly larger than the insulator with aging and a damp sheath, indicating that the polarization loss and conductivity loss played an important role in the temperature rise.

Wang et al. [9] investigated the heating of the composite insulators with different pollution levels under different levels of humidity. Surface contamination aggravated the abnormal heating of the HV side of the composite insulators, and the amplitude of the abnormal heating grew in proportion to the pollution level and surrounding humidity. Wang et al. [16] found that the interface defect between the core rod and sheath of a decay-like insulator can generate partial discharge, resulting in the abnormal temperature of the composite insulator. Additionally, the increase in resistive current caused by core rod degradation produced heat. Zeng et al. [17] discovered that the abnormal heating of the decay-like composite insulators was related to polarization loss, partial discharge, and resistance loss caused by degradation, especially partial discharge. Surface contamination resulted in abnormal heating at the HV end of the composite insulators, according to Edson G et al. [18]. Ma et al. [19] examined the influence law of wind speed and ambient temperature on the temperature rise and used support vector machine multivariate regression (SVR) analysis to establish a temperature compensation model. The acquired fault temperature and the existing interference parameters were used as the input of the model, and the compensation temperature after eliminating the interference was obtained from the output of the model. The results showed that the model had high prediction accuracy and good correction effect. Zhong et al. [20] established an equivalent circuit model of short samples and 500 kV decay-like composite insulators to analyze the contribution of conductance loss and polarization loss to the temperature rise.

Domestic and foreign researchers have studied the single effect of environmental temperature, humidity, and wind speed on the abnormal heating of composite insulators. However, the influence of environmental factors on the infrared temperature of different types of abnormal heating composite insulators has not been distinguished. Furthermore, different types of abnormal heating have not been compared and analyzed. In this study, three types of composite insulators with abnormal heating were tested by infrared temperature measurement under different environmental humidity and wind speed conditions. The aspects of heating range, heating shape, and temperature rise were used to analyze the heating state of various composite insulators.

## 2. Materials and Methods

### 2.1. Specimen

**Figure 1:**
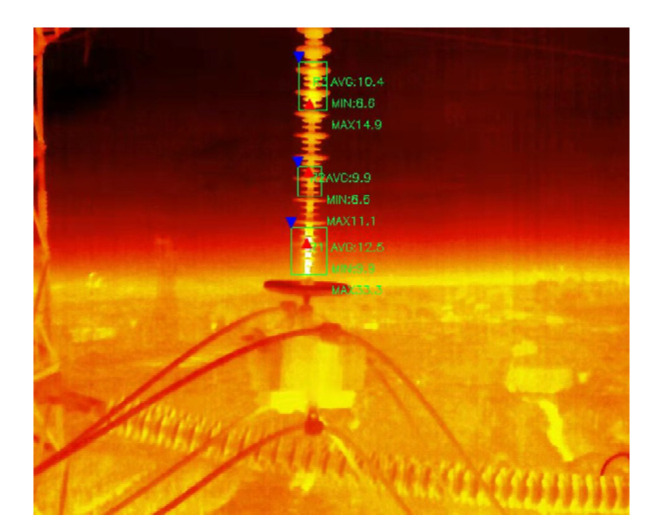
Infrared thermal image of the running composite insulator.

This study examined six 220 kV composite insulators with abnormal heating, including sheath-aging, decay-like, and contaminated insulators named A-1, A-2; B-1, B-2; and C-1, C-2, respectively. The sheath-aging insulators were from the same line. Due to the low probability of decay-like insulators, samples were collected from several transmission lines. The solid-coating method [21] was applied to construct the contaminated composite insulators artificially. The ratio of the equivalent salt deposit density (ESDD) to the non-soluble deposit density (NSDD) was 1:6, and the values for the ESDD were 0.08 mg/cm^2^ and 0.12 mg/cm^2^ [22]. Sodium chloride and diatomite were utilized to simulate conductive and insoluble substances, respectively. Figure 1 is the infrared thermal image of the running composite insulator.

**Figure 2:**
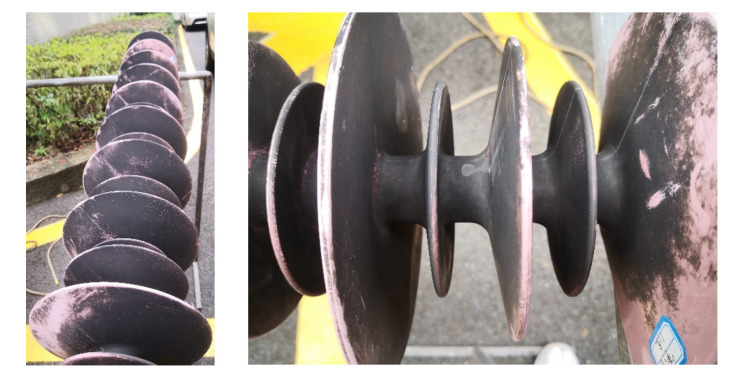
Visual inspection of sheath-aging insulator.

**Figure 3:**
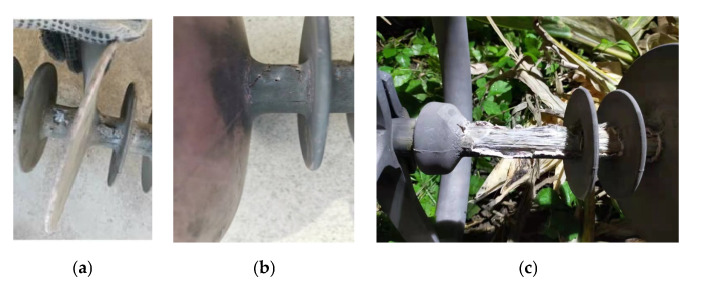
Visual inspection of decay-like composite insulator. (**a**) Cracks appear in the sheath; (**b**) Electrical erosion holes appear in the sheath; and (**c**) Bare core rod.

The appearance of the sheath-aging and decay-like composite insulators is shown in Figure 2 and Figure 3, respectively. There was obvious pulverization on the insulator sheds, and many cracks appeared on the pulverization layer. The severe aging of the sheds could be inferred from the depth of the cracks. The dirt and pulverization layers coexisted on the shed’s surface.

The appearance characteristics of the decay-like insulators can be classified as follows: (1) The sheath surface was black or had noticeable electrical erosion marks and even had electrical erosion holes; (2) The bonding strength of the core rod–sheath interface decreased and was even destroyed. (3) The surface color of the core rod gradually became black and even carbonized; (4) Filamentous fiber appears on the core rod surface, like decayed wood. The macroscopic characteristics of these insulators conformed to the definition of decay-like insulators [23].

### 2.2. Experimental Set-Up

**Figure 4:**
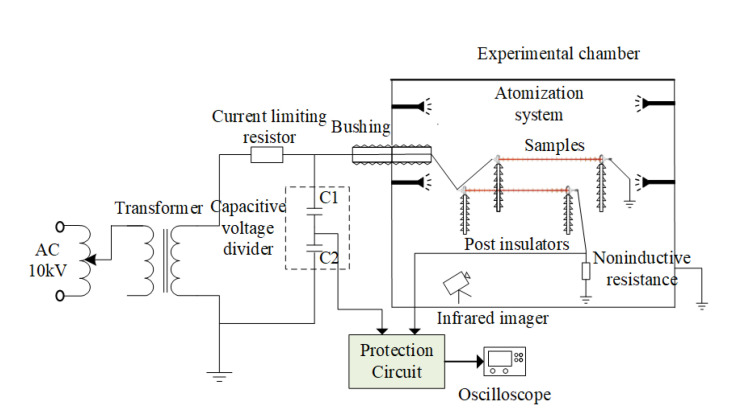
Schematic diagram of the experimental set-up.

In order to change humidity conveniently, the temperature characteristic test of abnormal heating composite insulators was carried out in the artificial climate chamber; the experimental layout is shown in Figure 4.

The applied voltage was measured by a capacitive voltage divider and voltmeter, and the ratio of the voltage divider was 1000:1. In this study, the digital oscilloscope (Tektronix DPO7354C, USA) was used to record the voltage waveform obtained from the low voltage arm of the divider. A non-inductive resistor with a resistance of 1 kΩ was connected in series between the low-voltage end of the composite insulator and the ground as a sampling resistor, and the leakage current (LC) waveform was captured via I-U transform. In order to prevent damage to the resistor caused by excessive leakage current, the two ends of the resistor were connected in parallel with the discharge tube for protection. The LC measurement circuit was placed in a metal shielding box to reduce electromagnetic interference in the environment. The coaxial cable was used for signal transmission. The shielding layer of the coaxial cable was attached to the metal shielding box and grounded by one end.

The measurement equipment included FLIR T660 infrared (IR) thermal imager (FLIR SYSTEMS, USA), CoroCAM^®^6D full-blind ultraviolet (UV) imager (UViRCO, South Africa), temperature and humidity transmitter, and high-precision handheld anemometer. Using infrared temperature rise to determine the operating status of composite insulators is currently the most commonly used method [24,25,26].

The infrared thermal imager’s resolution, accuracy, spatial resolution, and image frequency were 640 × 480 pixels, ±1 °C, 0.68 mrad, and 30 Hz, respectively, which could measure temperature ranges of −40–150 °C. The transmittance was 95%. The gain of the UV imager was set to 85% during the test. The UV sensitivity, working band, and visible light sensitivity were 3 × 10^−18^ watt/cm^2^, 240–280 nm, and 0.0004 Lux, respectively. The detection distance of these two devices was 3 m.

### 2.3. Experimental Process

#### 2.3.1. The Influence of Ambient Humidity

The wind speed in the artificial climate chamber was kept at 0 m/s, and the relative humidity was adjusted to 50%, 70%, and 90%, respectively. Since the ambient temperature during the test was stable at approximately 9–12 °C, the impact of ambient temperature could be ignored. The experimental steps were as follows:

(1) Sample arrangement. Due to the small size of the artificial chamber, the samples were placed horizontally on the post insulators. The infrared thermal imager was able to take a complete picture of the whole composite insulator and avoid the shelter of sheds when it was vertically suspended. Two abnormal heating composite insulators were conducted for each test, and the upper and lower two were A-1 and A-2, respectively.
(2) Environmental humidity adjustment. The humidity in the chamber was adjusted and evenly distributed using an ultrasonic fogger, fan, dehumidifier, and thermometer–hygrometer. Experiments were carried out from high to low humidity.
(3) Applied voltage. When the humidity reached the target value, the environment humidity was maintained for 1 h to wet the composite insulator thoroughly. The applied voltage was rated operating voltage, 1.1 × 220/√3 ≈ 140 kV. The composite insulator was performed for 1 h.
(4) Measurement. The infrared thermal imager was used to photograph abnormal heating, and the maximum temperature (*T_max_*) and the minimum (*T_min_*) of the composite insulator were recorded. The abnormal heating composite insulator’s temperature rise was Δ*T* = *T_max_* − *T_min_*, and the unit was K. The discharge of the insulator was observed by an ultraviolet imager, and the electrical voltage and leakage current were recorded by an oscilloscope.

#### 2.3.2. The Influence of Wind Speed

In order to explore the temperature characteristics of the insulator under various wind speeds, the ambient RH was maintained at 70%, and an AC voltage with an effective value (root mean square, RMS) of 140 kV was applied. The wind speed was adjusted to 0 m/s, 1 m/s, 2 m/s, and 3 m/s by an industrial fan and a governor.

## 3. Results

### 3.1. The Influence of Ambient Humidity

#### 3.1.1. Temperature Rise

**Figure 5:**
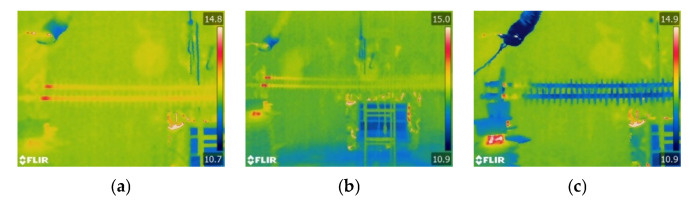
Infrared thermal images of sheath-aging insulators under different levels of humidity. (**a**) RH = 50%; (**b**) RH = 70%; and (**c**) RH = 90%.

The infrared thermal images of the sheath-aging insulator are displayed in Figure 5. The abnormal heating position is at the HV end, while the middle and low voltage (LV) end do not appear. When the relative humidity is 50%, the heating range of the composite insulator is from the HV end fitting to the first shed. When the relative humidity reaches 90%, there is abnormal heating between the first and second shed of the composite insulators, indicating that the increase in humidity causes a rise in the heating range. The temperature rise is less than 1 K after scraping 1–2 mm off the surface layer.

**Figure 6:**
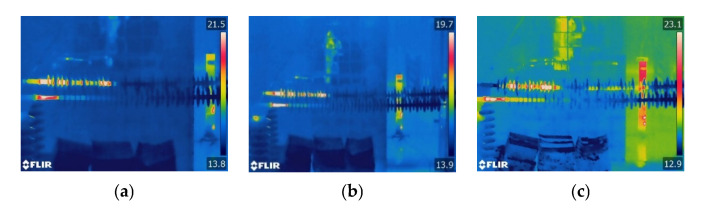
Infrared thermal images of decay-like insulators under different levels of humidity. (**a**) RH = 50%; (**b**) RH = 70%; and (**c**) RH = 90%.

The infrared thermal images of the decay-like composite insulators at various ambient humidity levels are shown in Figure 6. The decay-like insulator has a wide range of heating, which is much larger than the sheath-aging insulator. The heating shape of the decay-like insulator is regarded as segmental heating. With the increase in humidity, the heating range increases, and the central region of the decay-like insulator exhibits segmental heating, which may be caused by the wetting of the insulator and the growth of the leakage current. The high voltage side’s heating range is approximately 1 m when the RH is 90%.

**Figure 7:**
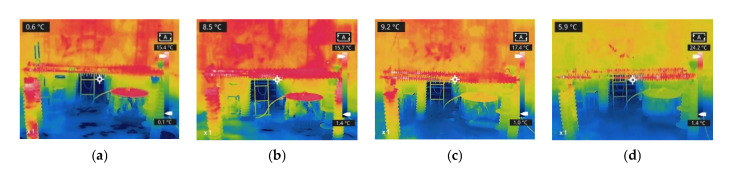
Infrared thermal images of contaminated insulators under different levels of humidity. (**a**) RH = 50%; (**b**) RH = 70%; (**c**) RH = 90%; and (**d**) RH = 100%.

The infrared images of the contaminated insulators at various environmental levels of humidity are shown in Figure 7. When the relative humidity is 70% or below, the heating zone of the polluted insulator is localized at the HV side. The composite insulator heats to varying degrees as the relative humidity reaches 90% and 100%. As the humidity increases to 90% and above, the pollution on the insulators becomes completely wet, and the leakage current increases sharply, producing heat. Under the same ambient humidity, the length of the heating range increases with the surface pollution of the composite insulators.

The heating range of decay-like and contaminated insulators is significantly longer than that of sheath-aging insulators, as seen by comparing the three abnormal heating types mentioned above. The heating range of the sheath-aging insulators is concentrated in the HV end to the second shed, whereas the HV end and middle part of the decay-like insulators both have abnormal heating. When the humidity is low, the heating range for contaminated insulators is primarily concentrated on the HV side, while the entire insulator may experience heating conditions under high humidity. From the perspective of heating shape, sheath-aging insulators can be regarded as point heating, and decay-like and polluted insulators can be considered segment heating.

**Figure 8:**
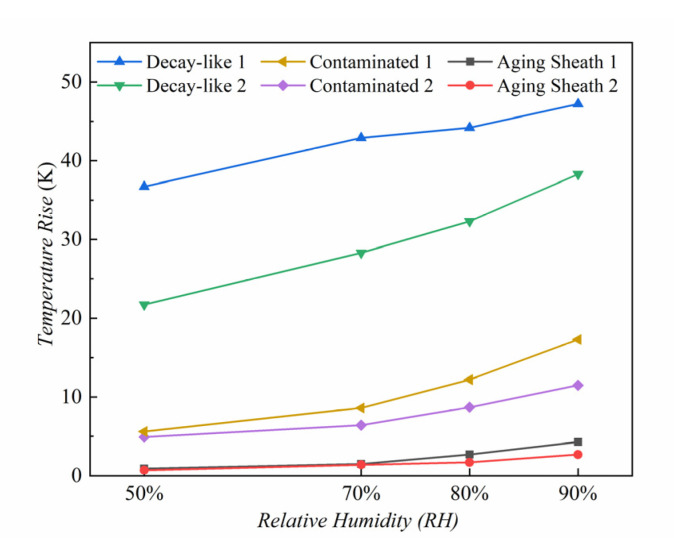
The Δ*T* of composite insulators under different levels of humidity.

Figure 8 depicts the temperature rise in the composite insulators with abnormal heating under various humidity levels.

When the relative humidity increases from 50% to 70%, the average Δ*T* of the sheath-aging insulators increases by 44.44%. The average Δ*T* rises by 52.21% as the RH rises from 70% to 90%. This discrepancy indicates that the ambient humidity greatly influences the Δ*T* of the sheath-aging insulators, and the Δ*T* increases obviously under high humidity.

According to Figure 8, the heating of the decay-like composite insulators is severe under the relative humidity of 50%, and the average Δ*T* reaches 29.2 K. Under low humidity, the Δ*T* of the sheath-aging insulator is less than 1 K, whereas the decay-like insulator heats obviously. As a result, the Δ*T* under low moisture is a critical characteristic that distinguishes the decay-like insulator from the sheath-aging insulator. The heating caused by polarization loss and leakage current under low humidity is negligible, indicating that the core rod’s defects are the root cause of its abnormal heating rather than the heating caused by the increase in ambient humidity. The Δ*T* of the composite insulators gradually rises as ambient moisture levels rise. The average Δ*T* of the decay-like insulator rises by 20.21% when the relative humidity increases from 50% to 70% and rises by 28.25% from 70% to 90%. The findings indicate that decay-like insulators are less affected by environmental humidity than are sheath-aging insulators.

It can be seen from Figure 8 that the Δ*T* of C-2 is higher than that of C-1 under the same ambient humidity, demonstrating that the Δ*T* increases with the ESDD. When the relative humidity is 50%, the average Δ*T* of the polluted insulator is approximately 5 K, which is higher than that of the sheath-aging insulator. In dry conditions, the surface pollution resistance of the composite insulator is high, and its leakage current is low, resulting in a low Δ*T*. The increase in humidity causes the water in the air to invade continuously the polluted layer, increasing the leakage current and the Δ*T*. The average Δ*T* of a contaminated insulator increases by 30% and 28.23%, respectively, as the relative humidity rises from 50% to 70% and from 70% to 90%. The rise in the polluted insulators remains consistent as environmental humidity rises, and the impact of environmental humidity on polluted insulators is likewise less than that of sheath-aging insulators. The Δ*T* increases by 27.43% when the relative humidity increases from 90% to 100%, even if the ambient humidity increases by 10%. This increase shows that when the air humidity reaches saturation, the surface of the composite insulator is entirely saturated, and abnormal heating is the most serious.

#### 3.1.2. Electrical Discharge

The 1 min video recorded by the ultraviolet imager is used to observe the discharge of the composite insulator. The number of ultraviolet photons every 1 s in the video is recorded, and then the average value is calculated. The number of average photons within 1 min at the major discharge position (MDP) is shown in Table 1. The shooting parameters are consistent. The unit of photons was a thousand.

It can be seen from Table 1 that the number of photons of different insulators under various conditions show the following characteristics:

As seen from the sheath-aging insulators, there is no obvious discharge at the HV end, but there exist some stray corona. With the increase in humidity, the stray corona increase slightly. Therefore, it is inferred that the abnormal heating of the sheath-aging insulator is not related to partial discharge.

The number of photons demonstrates the evident partial discharge on the decay-like insulator, and the heating of the decay-like insulator is the most serious at the severe partial discharge. The most severe heating is located close to the HV end sheath, as shown by the heating of decay-like insulators at RH = 70%. Apparent partial discharge occurs at the highest heating position, indicating that the partial discharge is the main reason for heating decay-like insulators. Due to the thermal effect of the partial discharge, the impact of the charged ions will not only deteriorate the insulating material but also raise the temperature of the partial discharge area. The field strength at the decay-like core rod increases significantly as the core rod ages, making it more likely to induce partial discharge than other portions. The heat generated by the partial discharge progressively accumulates, resulting in abnormal heating.

**Figure 9:**
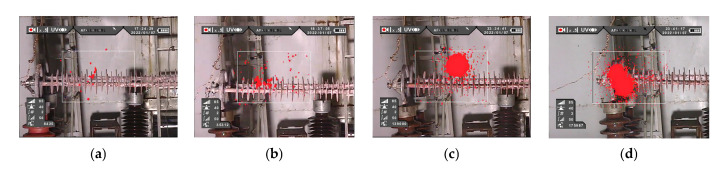
UV images of contaminated insulators under different levels of humidity. (**a**) RH = 50%; (**b**) RH = 70%; (**c**) RH = 90%; and (**d**) RH = 100%.

UV images of the contaminated insulator are shown in Figure 9. For the convenience of analysis, it is assumed that the shed next to the high-voltage end is the first piece and that it rises along the low-voltage end.

It can be seen from Figure 9 that under the relative humidity of 50%, there is a weak partial discharge between sheds 7–9 of the contaminated insulators. The partial discharge is enhanced when it reaches 70%. The sheath has abnormal heating, but it is not the most severe place of heating, indicating that partial discharge is one of the reasons for the contaminated insulator but not its primary cause. When RH = 100% for 1 min, there is no discharge on the surface of the contaminated insulator. The leakage current is increasing sharply as the pollution on the insulator surface becomes extensively wetted.

A dry zone appears between sheds 7–10 of the contaminated insulator after the test runs for 3 min, and a noticeable partial discharge is generated under the effect of the electric field. After 10 min, the sheath of the HV end of the contaminated insulator reveals a clear partial discharge, while the discharge between sheds 7–10 vanishes. Since then, the discharge area of the contaminated insulator is maintained at the HV end sheath. Under the relative humidity of 100%, the sheath of the HV end appears to be the most serious place on the infrared image, indicating that the partial discharge aggravates the abnormal heating of the contaminated insulator.

#### 3.1.3. Leakage Current

**Figure 10:**
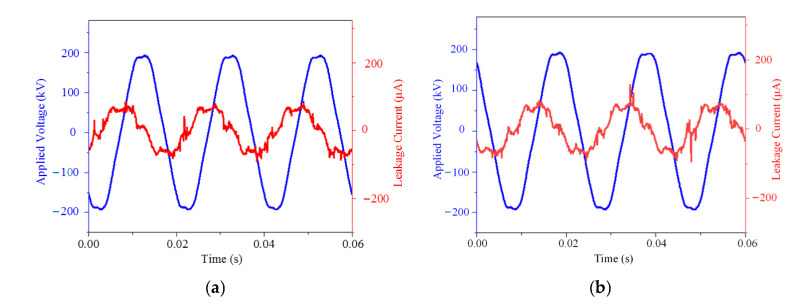
Applied voltage and LC waveforms of sheath-aging insulators. (**a**) A-1 and (**b**) A-2.

Figure 10 displays an experimental voltage and leakage current at a relative humidity of 70%. The fundamental component of the leakage current is extracted, and the phase difference between the fundamental component of the leakage current and the test voltage is calculated to reflect the phase characteristics of the leakage current. The fundamental effective value of leakage current and the phase difference are shown in Table 2. Due to the influence of stray corona and environmental noise, the LC waveform exhibits burrs.

The leakage current of the sheath-aging insulator is capacitive and still maintains good insulation performance, indicating that the leakage current is not the cause of the insulator with aging and damp sheath heating.

Due to the PD generated by the decay-like core rod, the LC waveform is severely distorted, showing a triangular wave shape. Leakage current is not the cause of the decay-like insulator heating, as evidenced by the fundamental wave’s effective value of the leakage current being less than 30 μA. The phase difference among the three insulators is reduced to below −70°, and the resistive current increases significantly. The reason may be related to the contamination of the composite insulator surface and the deterioration of the core rod.

Since the water in the pollution is continuously evaporated during the heating process of the composite insulator, the leakage current will continue to change. Due to the existence of the partial discharge, the leakage current waveform distortion becomes serious. The fundamental effective values of the leakage current of C-1 and C-2 indicate that the surface contamination is in a dry state. However, the resistive current rises and the phase difference falls as a result of the surface pollution.

### 3.2. The Influence of Wind Speed

**Figure 11:**
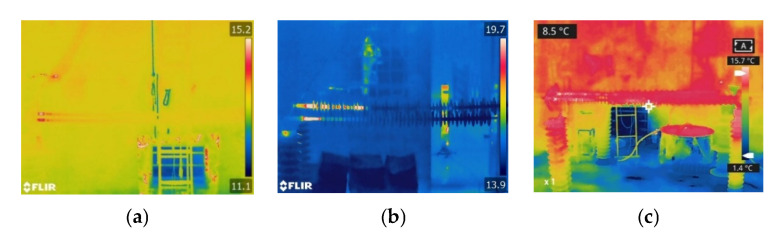
Infrared thermal images of different insulators under 0 m/s wind (RH = 50%). (**a**) sheath-aging; (**b**) decay-like; and (**c**) contaminated.

The results of three types of heating are shown in Figure 11. The heating position and length of sheath-aging and contaminated insulators do not change much during the increase in wind speed, so their temperature changes are directly described.

The sheath-aging composite insulators experience considerable heating from the HV end to the second shed when there is no wind. The heat between the first and second sheds weakens as the wind speed reaches 1 m/s. This phenomenon indicates that with the increase in wind speed, the heat of the sheath-aging insulators declines slightly and disappears.

As the wind speed increases, the heating range of the contaminated insulator steadily decreases. When the wind speed is 0 m/s, abnormal heat builds up between the C-1 HV fitting to shed 15 and the C-2 HV end fitting to shed 18. When the wind speed reaches 3 m/s, the heat between sheds 5–15 of C-1 and sheds 9–18 of C-2 disappears. With the gradual increase in wind speed, the temperature of the heating section of the contaminated insulator drops.

**Figure 12:**
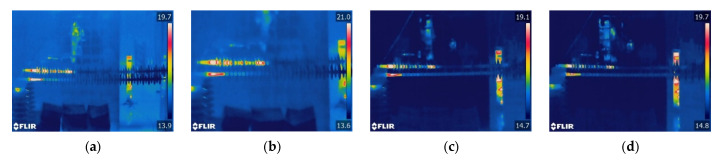
Infrared thermal images of decay-like insulators under different wind speeds. (**a**) v = 0 m/s; (**b**) v = 1 m/s; (**c**) v = 2 m/s; and (**d**) v = 3 m/s.

Figure 12 exhibits that the decay-like insulator’s heating range remains unaffected by the increase in wind speed. It can be seen that the temperature of the heating region gradually decreases with the wind speed. If the wind speed continues to increase, the abnormal heating in this area may disappear, resulting in a decrease in the heating range of the decay-like insulator.

The results of the Δ*T* with various wind speeds are as Table 3. As the wind speed increases from 0 m/s to 3 m/s, the average Δ*T* of the sheath-aging insulator declines by 73.33%. As can be observed, the Δ*T* of these insulators is easily affected by wind speed. When the ambient wind speed is 3 m/s, the Δ*T* decreases to 0.5 K or below, but in the absence of wind, the Δ*T* of the composite insulator is greater than 1 K. Therefore, the infrared temperature measurement of the composite insulator under windy conditions may cause missed or erroneous detection.

According to Table 3, the average Δ*T* of the decay-like insulator at 0 m/s is 35.6 K and is 19.55 K at 3 m/s. It can be seen that with the increase in ambient wind speed, the Δ*T* of decay-like insulators gradually decreases.

The Δ*T* of the contaminated insulator progressively decreases as the wind speed rises. When the wind speed increases from 0 m/s to 3 m/s, the average Δ*T* of the contaminated insulator decreases by 67.33%, which is between the sheath-aging insulator and the decay-like insulator. The decay-like insulator is less affected by the wind speed due to the higher Δ*T*, as can be seen from the table. The sheath-aging insulator with the smallest temperature rise is more likely to be overlooked by the wind speed, followed by the polluted insulator.

### 3.3. Microscopic Morphology

**Figure 13:**
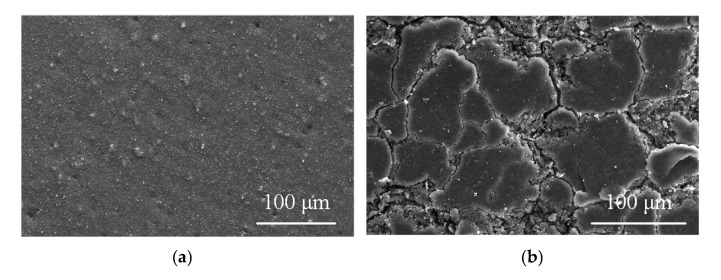
SEM images of the new and sheath-aging silicone rubber. (**a**) New and (**b**) Sheath-aging.

The previous experimental analysis and related research [10,27,28] show that the aging sheath of the high-voltage end of the composite insulator causes heating. The microstructure of the silicone rubber sheath is shown in Figure 13. In this study, the field emission scanning electron microscope (SEM) JSM-7800F (JEOL, Japan) is used to observe the microstructure. The samples are cleaned with anhydrous ethanol and then dried (50 °C, 24 h). The surface of the sample is sprayed with gold 150 s before observation.

**Figure 14:**
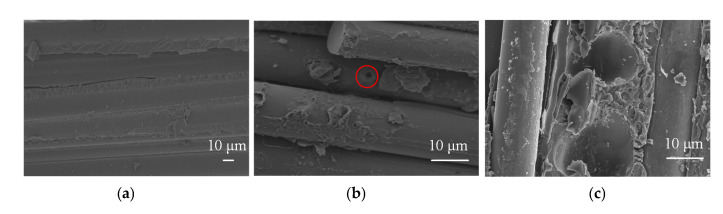
SEM images of the decay-like core rod on the surface of the heating area. (**a**) New; (**b**) Heating area (Bubbles are in the red circle); and (**c**) Heating area.

The surface layer of the aging silicone rubber is fragmented, indicating that its organic matter undergoes aging and cracking under long-term environmental factors. Furthermore, the internal pores are increased, which leads to easy infiltration and storage of water. Water is a polar molecule, leading to serious polarization heating. The microscopic morphology of the decay-like core rod is shown in Figure 14.

It can be seen from Figure 14 that the epoxy resin matrix of the core rod at the heating area is degraded, and the interface between the glass fiber and epoxy resin is destroyed. There are holes of different sizes on the surface, and it is speculated that partial discharge will lead to degradation and deterioration of the epoxy resin matrix. Due to the gradual invasion of water molecules during the moisture process of the core rod, its dielectric constant and dielectric loss factor gradually increase. In the high humidity environment, the dielectric constant of the decay-like rod increases greatly, and the electric field distortion of the composite insulator is more serious, which aggravates the partial discharge and makes the abnormal heating phenomenon of the decay-like composite insulator more serious [29].

## 4. Discussion

Under an alternating electric field, the composite insulator is heated due to moisture, and the main heat source is the polarization heating of dielectric conductivity and polarizable substances, especially water, inside the composite insulator. The heating power *p* can be characterized by the dielectric loss power of the material, namely:

(1) $$p_{1}=U_{d}^{2}\omega Ctan\delta$$

where *U_d_* represents the distribution voltage of the composite insulator, V; *ω* is the voltage angular frequency, rad/s; *C* is the equivalent capacitance of the composite insulator, F; and *tan* Δ is the dielectric loss factor at operating temperature.

With the increase in ambient humidity, the dipole polarization process of silicone rubber is intensified after absorbing water from the air, resulting in a gradual rise in its dielectric loss factor, which increases the dielectric loss of heating power caused by the polarization effect and produces abnormal heating. Under low humidity conditions, the abnormal Δ*T* of the insulators is caused mainly by internal insulation defects. With the increase in humidity, the proportion of insulator heating caused by moisture polarization increases to mask the abnormal Δ*T* caused by internal insulation defects. Therefore, under high humidity, the abnormal Δ*T* of the composite insulators may not accurately reflect the internal insulation defects of the composite insulators. The use of infrared temperature measurement under low humidity conditions is conducive to improving the detection accuracy of the composite insulators.

The increase in leakage current caused by the surface contamination is the main reason for the heating of the contaminated insulators. The heating power caused by the surface leakage current under the action of alternating electric field can be expressed by Equation (2):

(2) $$p_{2}=U_{d}I_{g}$$

where *U_d_* represents the distribution voltage of the composite insulator, V; *I_g_* is the resistive component of the leakage current of the composite insulator, *A*. The increase in *I_g_* will cause *U_d_* of the insulator to increase. Therefore, the heating power will be greatly increased, and the temperature difference of the composite insulator will gradually increase.

With the increase in wind speed, the temperature rises of the sheath-aging composite insulators gradually decrease. The convective heat transfer process between air and the composite insulator surface can be approximated by Newton’s cooling law, as shown in Equation (3):

(3) $$Q=qA=Ah(T_{w}-T_{f})$$

where *Q* is the heat transfer power and refers to the heat per unit time through the heat transfer surface, W; *A* is the heat transfer area expressed in m^2^; *h* is the convective heat transfer coefficient, W/(m^2^·K); and *T_w_* and *T_f_* are the surface temperature and fluid temperature of the object, K, where *T_w_* > *T_f_*.

Without considering the influence of radiation heat transfer coefficient, assuming that the wind direction is perpendicular to the central axis of the composite insulator, it can be approximately considered that the convective heat transfer coefficient *h* is proportional to the wind speed v, which can be expressed by Equation (4):

(4) h=5.0+4.7v

With the increase in wind speed, the convective heat transfer coefficient of the composite insulator sheath gradually increases, and the heat loss of air convection per unit of time increases, resulting in a gradual decrease in the temperature rise in the composite insulator.

According to the law of heat transfer, when the heat propagates from the inside to the outside, due to the heat transfer coefficient of the silicone rubber medium, certain dissipation will occur when the internal heat propagates to the silicone rubber sheath, so there is also a big difference between the internal and external temperatures. In addition, the heat loss caused by the external wind speed has little effect on the temperature of the inner rod of the sheath. The heat of the inner rod is high, so the high-temperature area is large. The heating length, value, and change rule of the three heating types are different, so it is an effective means to distinguish the heating types of the composite insulators by infrared detection when the environmental conditions are suitable [2,24,25,26].

If the insulators’ temperature rises appear in any of the following situations, they should be dealt with as soon as possible because the core rod may be decay-like (or there may be internal defects): (1) The point temperature rise in the high voltage end is greater than 15 K; (2) Segmental temperature rise occurs at the high voltage end (more than three sheds in length); or (3) The temperature rises of medium part and low voltage (more than 5 K). When using infrared to detect composite insulators, it is best to detect at low wind speed and low humidity to reduce the impact of the environmental factors. In addition, UV detection and leakage current online monitoring can be used to help distinguish the heating types.

## 5. Conclusions

In this paper, the influence of environmental factors (humidity and wind speed) on the infrared temperature measurement of different types of abnormal heating composite insulators is distinguished. Three types of defective insulators are distinguished from the abnormal temperature rise value and the heating range, and the causes of different types of abnormal heating are explored on the basis of the leakage current and microstructure.

(1) The silicone rubber’s deterioration and the humidity in the air are factors influencing the abnormal heating. Along with the increase in ambient humidity, the temperature rises, and the heating range of the three different types of abnormal heating insulators expands. The partial discharge brought on by the defects of the core rod is the primary cause of heating the decay-like insulator. The temperature rises of polluted insulators increases with the pollution level. The heating range of the contaminated and decay-like insulators is much longer than that of the sheath-aging insulators.
(2) The temperature rises of sheath-aging insulators are reduced or even eliminated under low humidity, but decay-like and contaminated insulators still heat significantly. As a result, a key characteristic distinguishing the decay-like and contaminated insulators from the sheath-aging insulators characterizes the heating phenomenon at low humidity.
(3) For the heating phenomenon caused by sheath loss, optimizing the electric field distribution is an effective control strategy. The configuration of the grading ring can be optimized by electric field simulation to reduce the electric field of the HV sheath.

## Figures and Tables

**Table 1:** Number of photons of different insulators under diverse conditions (Unit: thousand/k).

Samples	Average Number of Photons/(k)			MDP
	RH *=* 50%	RH *=* 70%	RH *=* 90%	
Sheath-aging	0.6	1.4	2.0	Middle part
Decay-like	55	97	112	HV end
Contaminated	8	35	67	Variable

**Table 2:** LC fundamental effective value and phase difference (RH = 70%).

Samples		Δ*T* (K)	Fundamental Effective Value (μA)	Phase Difference (°)
Sheath-aging	A-1	1.0	50.7	−86.2
	A-2	1.4	50.8	−84.1
Decay-like	B-1	42.9	29.6	−49.8
	B-2	28.3	20.2	−68.3
Contaminated	C-1	6.4	52.8	−58.7
	C-2	8.6	55.5	−66.3

**Table 3:** The Δ*T* of composite insulators under different wind speeds (RH = 70%).

Samples		Δ*T* (K)			
		0 m/s	1 m/s	2 m/s	3 m/s
Sheath-aging	A-1	1.5	1.0	0.7	0.4
	A-2	1.4	0.8	0.6	0.5
Decay-like	B-1	42.9	34.2	28.4	22.6
	B-2	28.3	23.2	19.5	16.5
Contaminated	C-1	6.4	4.6	3.6	2.3
	C-2	8.6	6.6	3.8	2.6

## Data Availability

Not applicable.
